# Supplementary figures and images for: Land cover drives large scale productivity-diversity relationships in Irish vascular plants
Source: PeerJ. 2019 May 31;7:e7035. doi: 10.7717/peerj.7035 (PMC6546085; doi:10.7717/peerj.7035)

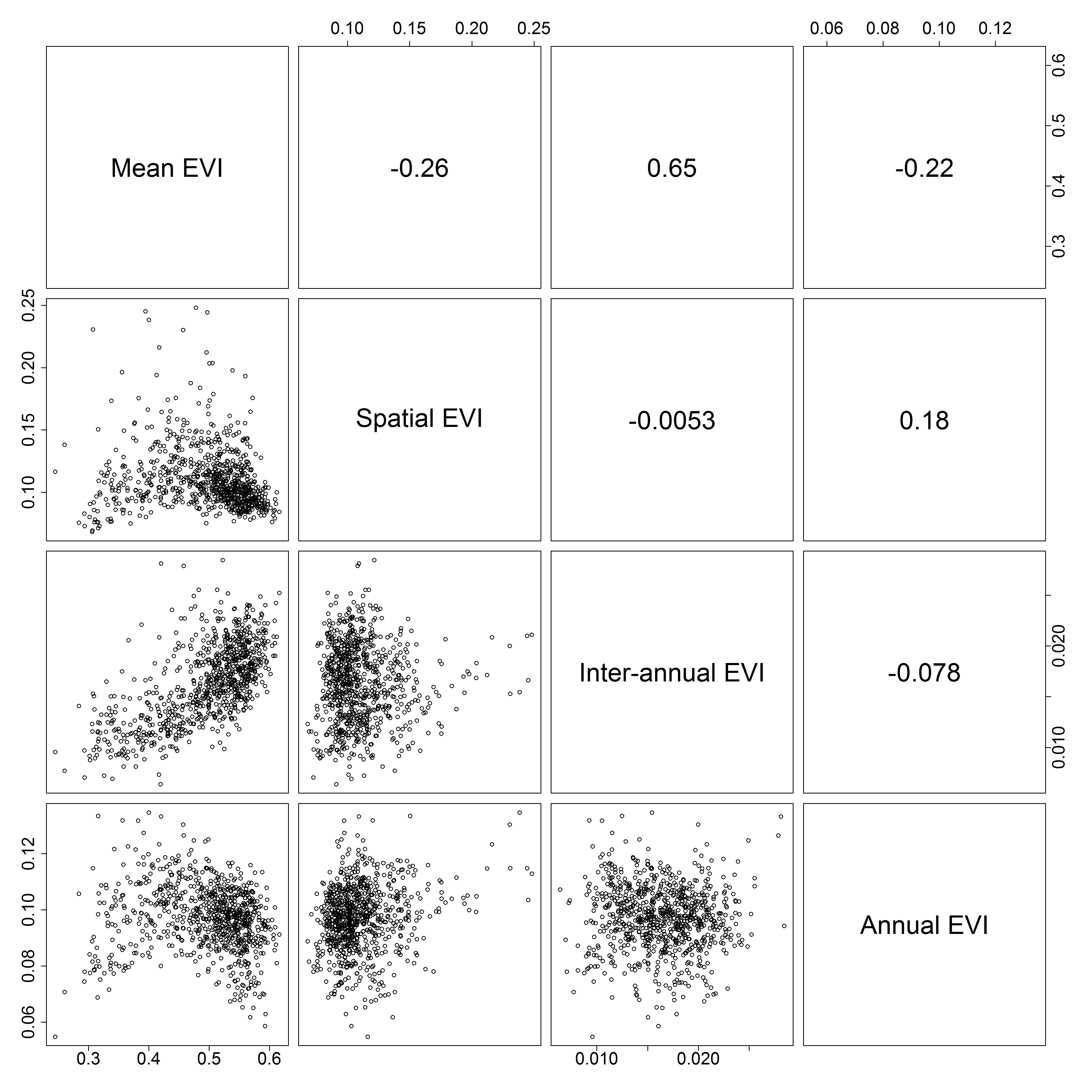

Supplement: Supplemental Information 1 — The relationship between the four EVI metrics and their Spearman’s correlations. All were significant at the 5% level except that between spatial standard deviation in EVI and inter-annual standard deviation in EVI. [file peerj-07-7035-s001.tiff]
