# Supplementary material for: Land cover drives large scale productivity-diversity relationships in Irish vascular plants
Source: PeerJ. 2019 May 31;7:e7035. doi: 10.7717/peerj.7035 (PMC6546085; doi:10.7717/peerj.7035)
Supplement: Supplemental Information 2 [file peerj-07-7035-s002.docx]

**Appendix 1: Species-area relationship of vascular plants**

The results of a power law species-area relationship between area of the hectad that was not pasture and corrected species richness fitted using a non-linear least squares model using the R package *nlme* where:

$S=cA^{z}$

A = area of non-pasture, S = corrected species richness of the hectad using FRESCALO, and c and z are estimated parameters. Starting values for parameter estimates were extracted from a linear regression in log-log space.


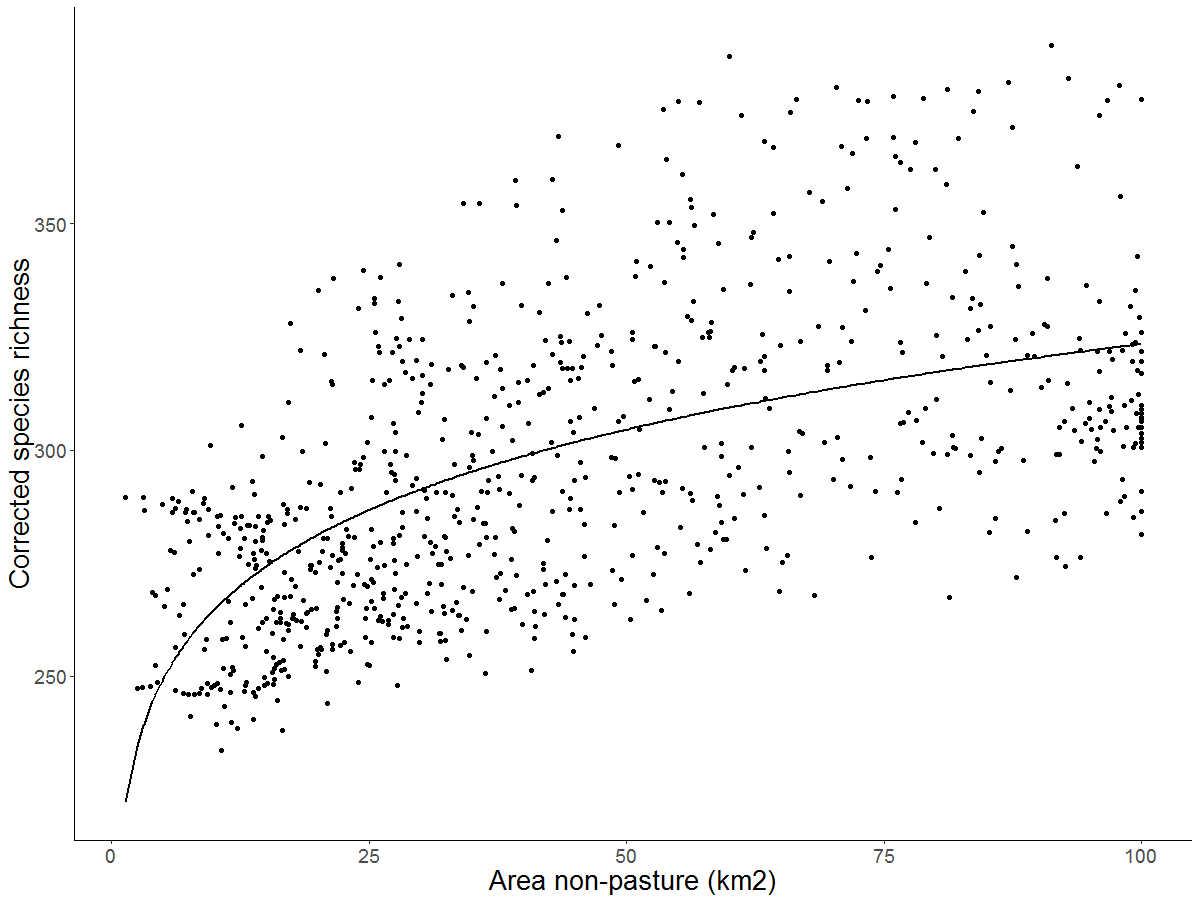


Parameter estimates:

c = 216.82 (standard error = 3.41, *P* < 0.001)

z = 0.09 (standard error = 0.004, *P* < 0.001)
